# A survey of personnel and services offered in 32 outpatient US clozapine clinics

**DOI:** 10.1186/s12888-021-03584-6

**Published:** 2021-11-19

**Authors:** Robert O. Cotes, Donna Rolin, Jonathan M. Meyer, Alexander S. Young, Amy N. Cohen, Tristan Gorrindo

**Affiliations:** 1 Department of Psychiatry and Behavioral Sciences, Emory University School of Medicine, Atlanta, USA; 2 University of Texas at Austin School of Nursing, Austin, USA; 3 Department of Psychiatry, University of California, San Diego, USA; 4 Department of Psychiatry, David Geffen School of Medicine at the University of California, Los Angeles, USA; 5 American Psychiatric Association, Washington, D.C., USA; 6 Optum Behavioral Care, Washington, D.C., USA

**Keywords:** Clozapine, Clozapine clinics, Interdisciplinary teams

## Abstract

**Background:**

Clozapine clinics can facilitate greater access to clozapine, but there is a paucity of data on their structure in the US.

**Methods:**

A 23-item survey was administered to participants recruited from the SMI Adviser Clozapine Center of Excellence listserv to understand characteristics of clozapine clinics.

**Results:**

Clozapine clinics (*N* = 32) had a median caseload of 45 (IQR = 21–88) patients and utilized a median of 5 (IQR = 4–6) interdisciplinary roles. The most common roles included psychiatrists (100%), pharmacists (65.6%), nurses (65.6%), psychiatric nurse practitioners (53.1%), and case managers (53.1%). The majority of clinics outreached to patients who were overdue for labs (78.1%) and had access to on-site phlebotomy (62.5%). Less than half had on call services (46.9%).

**Conclusions:**

In this first systematic description of clozapine clinics in the US, there was variation in the size, staffing, and services offered. These findings may serve as a window into configurations of clozapine teams.

**Supplementary Information:**

The online version contains supplementary material available at 10.1186/s12888-021-03584-6.

## Background

Clozapine is the most effective medication for treatment-resistant schizophrenia (TRS) [1], but it is underutilized in the US. In a study of Medicaid recipients with schizophrenia across 46 states, only 4.8% received clozapine [2]. Barriers for clozapine’s use can be divided into patient concerns (fear of side effects, need for frequent hematologic monitoring, transportation issues), prescriber concerns (limited knowledge and minimal time), and system/administrative issues (lack of organizational support, and insufficient coordination between inpatient and outpatient settings) [3–5]. Despite these barriers, clozapine clinics have been established in a variety of configurations and been successful serving a caseload of patients with TRS, in order to provide evidence-based care to those in need [3, 6, 7].

The Clinical Support System for Serious Mental Illness, also known as SMI Adviser, is a 5-year initiative funded by the Substance Abuse and Mental Health Services Administration and administered by the American Psychiatric Association. The objective of SMI Adviser is to support those caring for individuals with serious mental illness implement evidence-based and person-centered care. As part of this objective, SMI Adviser established a Clozapine Center of Excellence (CCOE) that aims to improve the lives of individuals with TRS by promoting the safe and effective use of clozapine. The CCOE realizes this aim by providing free accredited webinars and virtual learning collaboratives, on-demand consultations to individuals and communities, and clinical tips and resources. In addition, the CCOE includes an email listserv, which has established a network of clozapine providers throughout the US.

As a product of the CCOE network, there was an opportunity to further illustrate real-world characteristics of clozapine clinics. To our knowledge, there has never been a systematic evaluation of the characteristics of clozapine clinics in the US. Our objective was to conduct a survey to better understand the personnel, leadership, and services offered within clozapine clinics.

## Methods

A 23-item survey was administered to the SMI Adviser CCOE listserv in January 2020. As an incentive to participate, one survey completer was randomly selected to receive a copy of a new clozapine textbook. The survey was sent via email to the 250 subscribers of the listserv. The survey questions were developed by the SMI Adviser CCOE faculty and sought to understand a number of domains pertinent to clozapine clinics, including services offered and disciplines and roles within the treatment team. Participants were asked what changes they would most like to make to their existing practices, and what barriers were encountered in accomplishing these changes. A copy of the survey can be found in the Online Supplement.

Respondents were defined as working in a clozapine clinic if they met the following criteria: 1) they worked in an outpatient setting, 2) they managed more than one patient on clozapine, and 3) there were at least two staff of different disciplines on the clozapine team. Respondents who primarily practiced in inpatient, forensic, or residential treatment facilities were excluded from the analyses. Descriptive statistics were analyzed using SPSS version 26. For open-ended survey questions on what clinics would like to change and the barriers to accomplishing these changes, qualitative themes were identified and summarized. A Venn diagram using InteractiVenn [8] was used to show overlap between the five most common disciplines included on a clozapine clinic team. The methods were carried out in accordance with relevant guidelines and regulations. The American Psychiatric Association Institutional Review Board deemed the study minimal risk, waived the requirement for informed consent, and approved the methods of the study. The authors of the paper were given de-identified access to the survey data.

## Results

### Respondent demographics

The response rate was 55/250 (22%), of which 34 respondents met the criteria as being part of an outpatient clozapine clinic. There was a duplication of the description of two clinics, which included the same location and the same clinic characteristics, entered by different survey participants. For these clinics, the completed survey that included the most complete description of the clinic was utilized, and the other survey was discarded, leaving 32 unique clinics in the analysis. Of the respondents *N* = 24 (75%) were psychiatrists, *N* = 6 (18.8%) were pharmacists, *N* = 1 (3.1%) was a psychiatric nurse practitioner, and *N* = 1 (3.1%) was an internist. In terms of practice settings, *N* = 24 (75%) worked in a community mental health clinic, *N* = 3 (9.4%) worked in an academic institution, *N* = 3 (9.4%) worked in a VA setting, and *N* = 2 (6.3%) were in private practice.

### Clinic characteristics

The size of the clinics ranged from 3 to 200 patients on clozapine with a median of 45 (IQR = 21–88). Regarding the services offered in clozapine clinics, *N* = 25 (78.1%) offered outreach to patients overdue for labs, *N* = 20 (62.5%) had on-site phlebotomy, *N* = 15 (46.9%) had on-call services available after hours, *N* = 15 (46.9%) had on-site general medical care and consultation, *N* = 14 (43.8%) delivered group psychoeducation, *N* = 13 (40.6%) delivered medication to the patient, *N* = 12 (37.5%) used a protocol or guidelines to monitor side effects, and *N* = 7 (21.9%) offered group prescribing. Point-of-care absolute neutrophil count testing was used in *N* = 1 (3.1%) clinic.

The average clozapine clinic had a median of 5 (IQR = 4–6) distinct roles on the treatment team. The most common disciplines included on the team, in descending order were: psychiatrists (*N* = 32; 100%), pharmacists (*N* = 21; 65.6%), nurses (*N* = 21; 65.6%), psychiatric nurse practitioners (*N* = 17; 53.1%), case managers (*N* = 17; 53.1%), psychiatry residents (*N* = 14; 43.8%), social workers (*N* = 10; 31.3%), medical assistants (*N* = 6; 18.8%), medical students (*N* = 5; 15.6%), pharmacists with prescribing privileges (*N* = 4; 12.5%), psychologists (*N* = 4; 12.5%), primary care physicians (*N* = 3; 9.4%), administrative assistants (*N* = 3; 9.4%), peer specialists (*N* = 3; 9.4%), and physician assistants (*N* = 1; 3.1%).

**Fig. 1:**
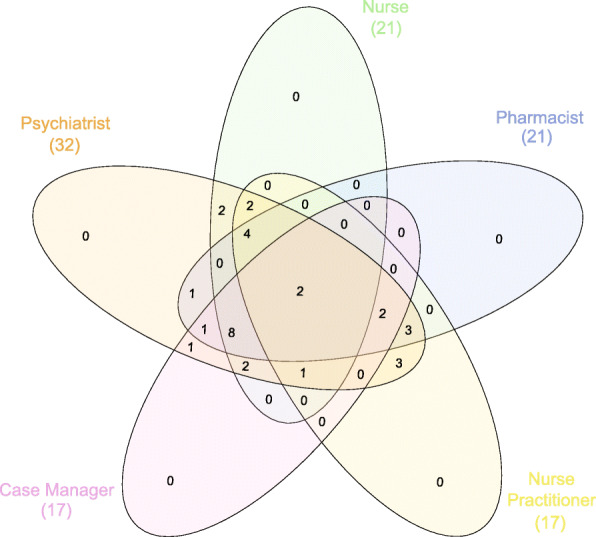
Venn diagram of clozapine staffing configurations

A Venn diagram of clozapine clinic staffing configurations is shown in Fig. 1 using the five most common disciplines. All five disciplines were used by *N* = 2 (6.3%) clinics, and *N* = 15 (46.9%) used a combination of four of the five disciplines. The most common configurations included 1) psychiatrist, nurse, pharmacist, and case manager (*N* = 8; 25%), 2) psychiatrist, nurse practitioner, nurse, and pharmacist (*N* = 4; 12.5%), 3) psychiatrist, nurse practitioner and pharmacist (*N* = 3; 9.4%), and 4) psychiatrist and nurse practitioner (*N* = 3; 9.4%). Either a nurse or a nurse practitioner was a member of the team in *N* = 30 (93.8%) clinics. Over three-fifths of clinics (*N* = 21; 65.6%) had a pharmacist on the team. Over three-fifths (*N* = 20; 62.5%) of clinics utilized either a case manager or social worker. Almost half of the clinics (*N* = 14; 43.8%) involved psychiatry residents. Few clinics (*N* = 3; 9.4%) had dedicated administrative or clerical support, and those that did were part of larger programs.

Respondents identified who were most likely to enter absolute neutrophil count values into clozapine REMS; top four were: pharmacists (*N* = 14; 43.8%), nurses (*N* = 13; 40.6%), physicians (*N* = 11; 34.4%), and clerical/administrative staff (*N* = 5; 15.6%). Respondents (*N* = 30; two respondents did not complete this section) on teams identified the following members of the interdisciplinary team as the leader of the clozapine clinic: psychiatrists (*N* = 19; 63.3%), pharmacists (*N* = 4; 13.3%), no designated leader (*N* = 3; 10%), nurses (*N* = 2; 6.7%), nurse practitioners (*N* = 1; 3.3%), and joint leadership between a psychiatrist and pharmacist (*N* = 1; 3.3%).

### Opportunities for improvement and barriers

When asked what respondents (*N* = 28) would like to change or include within their current system, the most common responses were (from more to less frequent): standardized group psychoeducation (*N* = 8; 26.8%), a protocol-driven system to manage side effects (*N* = 7; 25%), increasing their capacity to prescribe clozapine to more patients (*N* = 4;14.3%), utilizing point of care clozapine testing (*N* = 3; 10.7%), adding on-site phlebotomy (*N* = 3; 10.7%), increasing case management and social support services (*N* = 3; 10.7%), increasing wellness/exercise activities (*N* = 3; 10.7%), and integrating labs into the EHR (*N* = 3; 10.7%). When asked about the main barriers to change (*N* = 29 respondents), the most common responses were inadequate staffing (*N* = 8; 27.6%), insufficient time to enact the changes (*N* = 7; 24.1%), cost/financial issues (*N* = 7; 24.1%), institutional barriers (*N* = 5; 17.2%), EHR issues (*N* = 3; 10.3%), and a lack of medical leadership support (*N* = 2; 6.9%).

## Conclusions

**Fig. 2:**
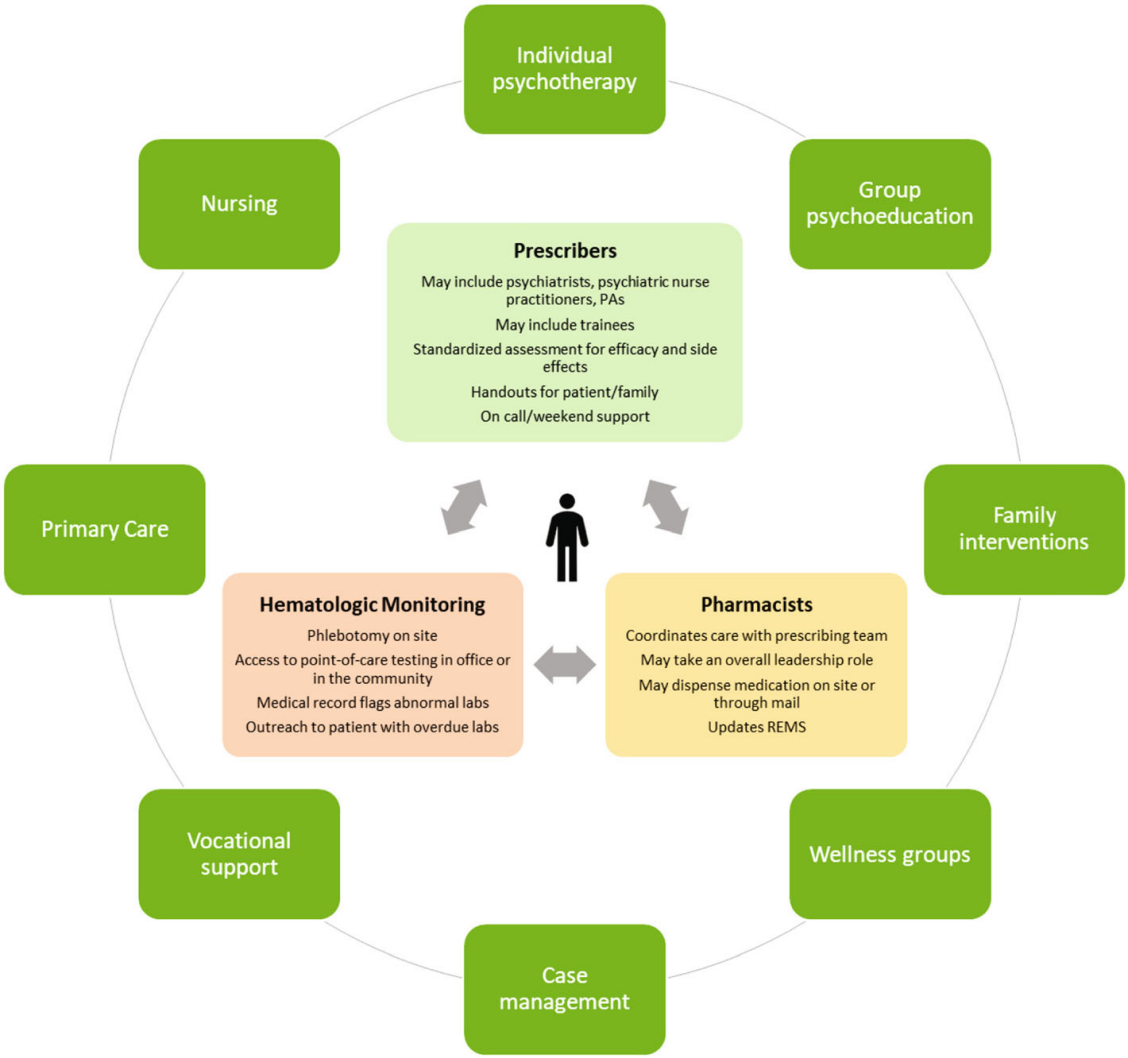
Representation of Services, Roles, and Care Coordination in a Clozapine Clinic

These results provide an important glimpse into the inner workings of clozapine clinics in the US. To our knowledge, this is the first systematic examination of the personnel, services, and configurations of outpatient clozapine clinics. The sample represented diverse practice settings and included community mental health sites, academic institutions, VA clinics, and private practices. Since a lack of clinic infrastructure is a barrier to widening clozapine use [6], a picture of various configurations to deliver and manage clozapine for a cohort of individuals with TRS may be of value to clinicians, administrators, and health care systems looking to expand clozapine use. Psychiatrists, nurses, pharmacists, nurse practitioners, and case managers were often key team members in clozapine clinics. Psychiatry residents were participants in just under half of clinics, which bodes well for the future of clozapine prescribing. Robust training curricula have been implemented within clozapine clinics and may be an important opportunity for learners to develop confidence using clozapine with experienced mentors [9]. The variation in personnel configurations is assumed to be in response to available personnel and/or developed resources or external collaborations to meet the needs of their patient communities. Based on the survey results, Fig. 2 depicts a framework for a clozapine clinic that can be adapted to an individual clinic setting.

Survey respondents identified multiple opportunities for their clinics to improve. Developing standardized protocols to manage side effects and offering group psychoeducation were common responses of areas to augment current programming. The personnel configurations mentioned by the survey respondents did not necessarily indicate the clinic felt that the staffing was ideal. About a quarter of the sample felt their current staffing was a barrier to accomplishing the changes they would like to implement. Peer specialists were rarely included in the sample, but could be important additions to many teams, as peer support for people with serious mental illness is associated with recovery, hope, and empowerment [10]. Clozapine teams rarely included psychologists, who can play a key role delivering evidence-based treatments such as cognitive behavioral therapy for psychosis (CBT-P), an intervention that may be of benefit to some with TRS [11]. It is possible that other disciplines were able to perform these psychotherapeutic interventions. Point of care testing was only used in one clinic, but may be an important tool to increase access to clozapine [12]. Low utilization of point of care testing may be function of this technology only recently being more widely available at the time of the survey. Furthermore, the use of standardized protocols for side effect and efficacy monitoring were rare.

The study has several limitations, including a small sample size. Respondents were all part of the clozapine COE listserv, which only represents a subset of US clozapine clinics. We were unable to ascertain from the survey the amount of effort allocated to each discipline (for example, it was unclear if the psychiatrist was full or part-time, or if there were one or two social workers in the clinic). It is also possible that some clozapine clinics may have access to other resources, such as psychology or case management, that were not formally a part of the clozapine clinic team. It was also not known if there were systems in place for the various disciplines to coordinate care within the team.

These results may be helpful in guiding organizations to create clozapine programs or providing ideas for existing clinics to expand. Future research on clozapine clinics may link different personnel configurations to patient outcomes (symptoms, side effects, quality of life) and cost. In depth qualitative interviews among clinic leaders could be utilized to better understand barriers. Feedback from individuals that receive care in clozapine clinics may be helpful to improve the patient experience and further identify gaps in current services. Organized team approaches have been utilized with success in coordinated specialty care models for individuals with early psychosis [13], and similar models could be adapted for clozapine clinics.

## Supplementary Information

**Additional file 1.**

## Data Availability

The dataset used during the current study is available from the corresponding author on reasonable request.
