# Supplementary material for: A survey of personnel and services offered in 32 outpatient US clozapine clinics
Source: BMC Psychiatry. 2021 Nov 19;21:583. doi: 10.1186/s12888-021-03584-6 (PMC8605520; doi:10.1186/s12888-021-03584-6)
Supplement: Supplementary file 1 — Additional file 1. [file 12888_2021_3584_MOESM1_ESM.pdf]

Clozapine System Questionnaire

1. Name:

2. Profession:

- [ ] Psychiatrist
- [ ] Other Physician
- [ ] Nurse Practitioner
- [ ] Other Nurse
- [ ] Psychologist
- [ ] Social Worker
- [ ] Pharmacist
- [ ] Other (specify):

*

3. Email:

4. Practice location:

5. Type of practice: (choose all settings in which you administer clozapine)

If you administer clozapine in multiple settings, respond first for your largest clozapine system. If you want to report on multiple settings, you can do the survey more than once.

- [ ] Community mental health clinic
- [ ] Academic or university setting
- [ ] Private practice
- [ ] Partial hospital or intensive outpatient program
- [ ] Inpatient or hospital
- [ ] Residential facility
- [ ] Jail or prison
- [ ] Other (specify):

*

---

6. Approximately how many patients are currently prescribed clozapine by you personally or in systems that you lead?

If you work in multiple clozapine settings, enter your total across all settings.

Which functions are your systems currently providing?

7. Lab-related:

- ☐ On-site phlebotomy
- ☐ Reminders to patients when due for labs
- ☐ Outreach to patients when overdue for labs
- ☐ Alerts to clinician when patient is overdue for labs
- ☐ Use of point-of-care ANC (absolute neutrophil count) testing device
- ☐ Other (specify):

  *

8. On-call service for clozapine-related issues:

- ☐ After hours
- ☐ Weekends
- ☐ None provided

9. Medical care:

- ☐ On-site general medical care and consultation
- ☐ Vital sign monitoring
- ☐ Other (specify):

  *

10. Delivery of services in group format:

- [ ] Psychoeducation
- [ ] Physical health screening
- [ ] Group prescribing (medication group)
- [ ] Other (specify):

*

11. Electronic health record (EHR) related:

- [ ] Use of a population health registry for clozapine patients
- [ ] Mechanism to identify clozapine-eligible patients within the EHR
- [ ] Mechanism to identify patients prescribed clozapine within the EHR
- [ ] Clozapine order sets for laboratory monitoring or prescriptions or associated orders
- [ ] Clozapine note templates for progress notes or other documentation
- [ ] Lab ordering and reporting
- [ ] Other (specify):

*

12. Side-effect monitoring:

- [ ] Protocol or guidelines for monitoring side effects
- [ ] Structured screening questionnaires for monitoring side effects
- [ ] Protocols for managing specific side effects
- [ ] Other (specify):

*

13. Monitoring treatment response:

- [ ] Protocol or guidelines for monitoring treatment response
- [ ] Structured symptom assessment (specify): *
- [ ] Structured functional assessment (specify): *

14. Individual or group interventions to mitigate metabolic side effects:

- [ ] Nutritionist
- [ ] Group psychoeducation on health promotion/diet/exercise support
- [ ] Other (specify): *

15. Pharmacy:

- [ ] On-site pharmacy
- [ ] Medication delivered to clinic
- [ ] Medication delivered to patient
- [ ] Medication pick-up at pharmacy
- [ ] Other (specify): *

16. Telehealth (specify):

17. Case management (specify):

18. Patient education:

- [ ] Handouts
- [ ] Curricula
- [ ] Materials
- [ ] Website
- [ ] Other (specify):

*

19. Family support and education:

- [ ] Family liaison
- [ ] Family meetings
- [ ] Family education handouts, curricula, materials, web site (specify):

*

- [ ] Other (specify):

*

(untitled)

20. Select people who play an active role in the clozapine system:

- [ ] Psychiatrist
- [ ] Physician (other)
- [ ] Psychiatric nurse practitioner
- [ ] Nurse (RN, LVN/LPN)
- [ ] Medical assistant
- [ ] Pharmacist with prescribing privileges
- [ ] Pharmacist
- [ ] Social worker
- [ ] Case manager
- [ ] Psychologist
- [ ] Administrative assistant
- [ ] Peer specialist
- [ ] Psychiatry residents
- [ ] Medical students or other trainees: specify

*

- [ ] Other (specify):

*

21. Who enters clozapine REMS ANC values online?

- [ ] Psychiatrist
- [ ] Other physician
- [ ] Psychiatric nurse practitioner
- [ ] Other nurse
- [ ] Pharmacist
- [ ] Clerical staff
- [ ] Point of care ANC testing device
- [ ] Other (specify):

*

(untitled)

22. What's the leadership of your clozapine system?

- [ ] Led by physician
- [ ] Led by psychiatric nurse practitioner
- [ ] Led by other nurse
- [ ] Led by pharmacist
- [ ] Other (specify):

*

23. What would you most like to change/include/expand within your current system?

24. What are the main barriers to accomplishing these changes?
